# Erratum for Kweon et al., “Substrate Specificity and Structural Characteristics of the Novel Rieske Nonheme Iron Aromatic Ring-Hydroxylating Oxygenases NidAB and NidA3B3 from Mycobacterium vanbaalenii PYR-1”

**DOI:** 10.1128/mBio.01872-20

**Published:** 2020-08-25

**Authors:** Ohgew Kweon, Seong-Jae Kim, James P. Freeman, Jaekyeong Song, Songjoon Baek, Carl E. Cerniglia

**Affiliations:** a Division of Microbiology, National Center for Toxicological Research, U.S. Food and Drug Administration, Jefferson, Arkansas, USA; b Division of Biochemical Toxicology, National Center for Toxicological Research, U.S. Food and Drug Administration, Jefferson, Arkansas, USA; c Division of Biosafety, National Institute of Agricultural Biotechnology, Suwon, Republic of Korea; d Laboratory of Receptor Biology and Gene Expression, National Cancer Institute, NIH, Bethesda, Maryland, USA

## ERRATUM

Volume 1, issue 2, e00135-10, 2010, https://doi:10.1128/mBio.00135-10. The files for Fig. S1 and S2 were incorrect (they were reversed). The files have been corrected online.

